# Biodistribution of Poly(alkyl cyanoacrylate) Nanoparticles in Mice and Effect on Tumor Infiltration of Macrophages into a Patient-Derived Breast Cancer Xenograft

**DOI:** 10.3390/nano11051140

**Published:** 2021-04-28

**Authors:** Abhilash D. Pandya, Tore-Geir Iversen, Siver Moestue, Maria T. Grinde, Ýrr Mørch, Sofie Snipstad, Andreas K. O. Åslund, Geir F. Øy, Wanja Kildal, Olav Engebråten, Kirsten Sandvig, Tore Skotland, Gunhild M. Mælandsmo

**Affiliations:** 1 Department of Tumor Biology, Institute for Cancer Research, Oslo University Hospital, The Norwegian Radium Hospital, 0379 Oslo, Norway; Abhilash.Pandya@rr-research.no (A.D.P.); Geir.Frode.Oy@rr-research.no (G.F.Ø.); olav.engebraten@medisin.uio.no (O.E.); 2 Department of Molecular Cell Biology, Institute for Cancer Research, Oslo University Hospital, The Norwegian Radium Hospital, 0379 Oslo, Norway; tore-geir.iversen@rr-research.no (T.-G.I.); ksandvig@ibv.uio.no (K.S.); 3 Department of Clinical and Molecular Medicine, Norwegian University of Science and Technology, 7491 Trondheim, Norway; siver.a.moestue@ntnu.no; 4 Department of Circulation and Medical Imaging, Norwegian University of Science and Technology, 7491 Trondheim, Norway; maria.t.grinde@ntnu.no; 5 SINTEF AS, Department of Biotechnology and Nanomedicine, 7034 Trondheim, Norway; yrr.morch@sintef.no (Ý.M.); sofie.snipstad@sintef.no (S.S.); andreas.aaslund@sintef.no (A.K.O.Å.); 6 Department of Physics, Norwegian University of Science and Technology, 7491 Trondheim, Norway; 7 Cancer Clinic, St. Olav’s Hospital, 7030 Trondheim, Norway; 8 Institute for Cancer Genetics and Informatics, Oslo University Hospital, The Norwegian Radium Hospital, 0379 Oslo, Norway; wki@ous-hf.no; 9 Department of Oncology, Oslo University Hospital, 0450 Oslo, Norway; 10 Institute of Clinical Medicine, Medical Faculty, University of Oslo, 0315 Oslo, Norway; 11 Department of Biosciences, University of Oslo, 0315 Oslo, Norway; 12 Department of Medical Biology, University of Tromsø, 9019 Tromsø, Norway

**Keywords:** poly(alkyl cyanoacrylate) nanoparticles, breast cancer, biodistribution, macrophage infiltration, magnetic resonance spectroscopy

## Abstract

We have investigated the biodistribution and tumor macrophage infiltration after intravenous injection of the poly(alkyl cyanoacrylate) nanoparticles (NPs): PEBCA (poly(2-ethyl-butyl cyanoacrylate), PBCA (poly(n-butyl cyanoacrylate), and POCA (poly(octyl cyanoacrylate), in mice. These NPs are structurally similar, have similar PEGylation, and have previously been shown to give large variations in cellular responses in vitro. The PEBCA NPs had the highest uptake both in the patient-derived breast cancer xenograft MAS98.12 and in lymph nodes, and therefore, they are the most promising of these NPs for delivery of cancer drugs. High-resolution magic angle spinning magnetic resonance (HR MAS MR) spectroscopy did not reveal any differences in the metabolic profiles of tumors following injection of the NPs, but the PEBCA NPs resulted in higher tumor infiltration of the anti-tumorigenic M1 macrophages than obtained with the two other NPs. The PEBCA NPs also increased the ratio of M1/M2 (anti-tumorigenic/pro-tumorigenic) macrophages in the tumors, suggesting that these NPs might be used both as a vehicle for drug delivery and to modulate the immune response in favor of enhanced therapeutic effects.

## 1. Introduction

Although chemotherapy during recent years has improved the treatment and prognosis of different cancers, there are still challenges with severe adverse effects, drug resistance, and an insufficient therapeutic effect in several cancer types. The use of drug-loaded nanoparticles (NPs) could improve cancer therapy. Several such products have reached the market, and many new product candidates are presently in clinical trials [1]. The challenges and opportunities of using NPs for cancer drug delivery have been discussed in several reviews [2,3]. One advantage of using NPs for drug delivery is linked to the trapping in tumors due to the enhanced permeability and retention (EPR) effect [4], although the importance of this effect is being discussed [5,6]. Furthermore, the inclusion of drugs in NPs can give a reduced systemic toxicity [7] and induce synergistic effects from the simultaneous delivery of two or more drugs [8]. Drug encapsulation also allows for the enhanced solubility of poorly soluble drugs, protection of the drug, prolonged and controlled release, and altered biodistribution.

Poly(alkyl cyanoacrylate) (PACA) was initially developed as surgical glue; it was later demonstrated to be an interesting drug carrier, as it both allowed high drug-loading capacity and was biodegradable [9]. We have shown that the alkyl chain of the cyanoacrylate monomer is important for the rate of degradation of the PACA NPs [10]. Furthermore, we demonstrated by using several cell lines that the cytotoxicity was dependent on the monomer used; i.e., poly(2-ethyl-butyl cyanoacrylate) (PEBCA) NPs were less toxic than poly(n-butyl cyanoacrylate) (PBCA) NPs, which were less toxic than poly(octyl cyanoacrylate) (POCA) NPs [11]. Recently, we showed that these three PACAs, when incubated with the breast cancer cell lines MCF-7, MDA-MB-231, and MDA-MB-468 affected signaling factors (involved in ER stress and redox imbalance) and cell fate differently, despite being very similar in chemical composition, size, and charge [12]. Finally, we have shown that these three PACA NPs, having such subtle variations in NP structure, also had different impacts on autophagy when exposed to different breast cancer cell lines [13].

The PEBCA NPs loaded with the FDA-approved cytotoxic drug, cabazitaxel, i.e., a semisynthetic derivative of taxane that inhibits microtubule disassembly [14], had a therapeutic effect that outperformed free cabazitaxel in a patient-derived breast cancer xenograft in mice [15]. In that study, we quantified the biodistribution of the PEBCA NPs labeled with the fluorescent dye NR668, and we reported data suggesting the infiltration of anti-tumorigenic macrophages to be essential for the therapeutic effect. Based on the cell studies described above, showing large variations in cellular responses for the three types of PACA NPs, we decided to study the biodistribution and tumor macrophage infiltration after intravenous (i.v.) injection of PEBCA, PBCA, and POCA NPs. As shown in the present work, there are large differences in the biodistribution of the three PACAs, and their effects on tumor macrophage infiltration also differ. High-resolution magic angle spinning magnetic resonance (HR MAS MR) spectroscopy of tumor slices obtained after injection of PEBCA and PBCA NPs are also described.

## 2. Materials and Methods

### 2.1. Synthesis and Characterization of Nanoparticles

The PEGylated PACA NPs were synthesized by miniemulsion polymerization. An oil phase consisting of 2.5 g n-butyl cyanoacrylate, 2-ethyl-butyl cyanoacrylate, or octyl cyanoacrylate (monomer, Cuantum Medical Cosmetics, Barcelona, Spain) containing 0.3% (*w*/*w*) butylated hydroxytoluene (Fluka, Buchs, Switzerland), 10% (*w*/*w*) vanillin (Sigma-Aldrich, St. Louis, MO, USA), and 2% (*w/w*) Miglyol 812 (Cremer, Cincinnati, OH, USA) was prepared. Fluorescent particles for optical imaging were prepared by adding NR668 (modified Nile Red) [16], custom synthesis, 0.2% (*w*/*w*) to the oil phase.

An aqueous phase consisting of 0.1 M HCl (20 mL) containing Pluronic F68 (2 mM, Sigma, St. Louis, MO, USA) and Kolliphor HS15 (6 mM, Sigma, Munich, Germany) was added to the oil phase and immediately sonicated for 3 min on ice (6 × 30 s intervals, 60% amplitude, Branson Ultrasonics digital sonifier 450, Branson Ultrasonics, Danbury, CT, USA). The solution was rotated (15 rpm, SB3 rotator, Stuart, Stone, ST, UK) overnight at a room temperature before adjusting the pH to 5.0, using 1 M NaOH. The polymerization was continued for 5 h at room temperature on rotation. The dispersion was dialyzed (Spectra/Por dialysis membrane MWCO 100,000 Da, Spectrum Labs, San Francisco, CA, USA) against 1 mM HCl to remove unreacted PEG. The size (z-average (z-avg.) or number mean (n-mean), polydispersity index (PDI) and the zeta potential of the NPs in phosphate buffer (10 mM, pH 7.0) were measured by dynamic light scattering (DLS) and laser Doppler Micro-electrophoresis using a Zetasizer Nano ZS (Malvern Instruments, Malvern, WR, UK) and by nanoparticle tracking analysis (NTA) using NanoSight NS300 (Malvern Instruments, Malvern, WR, UK).

To estimate the size change of the NPs following the binding of human plasma proteins, NR668-containing NPs were added to citrated plasma and analyzed by NTA in fluorescent mode (excitation 488 nm, detection @500 nm long pass filter). The viscosity of plasma was set to 1.6 cP at 25 °C [17]. Plasma from three consenting donors was bought from St. Olav’s Hospital, Trondheim, Norway and was pooled before use (approval for SITEF AS: REK 2019/1080).

### 2.2. PEG Quantification

The PEGylation of NPs was quantified by ^1^H nuclear magnetic resonance (NMR), as previously described [18]. Briefly, a Bruker 400 MHz Avance III HD equipped with a 5-mm SmartProbe z-gradient probe and SampleCase (parameters: zg30 pulse sequence, 30° pulse, 1 s delay time, 65,536 points spectral width, 3.96 min acquisition time) was used. Preceding NMR, the dialyzed NPs were centrifuged, the supernatant was removed, and the particles were resuspended with deionized water before a second centrifugation step followed by drying of the pellet at 50 °C for 12−18 h. The sample was dissolved in acetone-d6. The spectra were processed using Spectres (v. 2019.1.0) with automatic baseline and phase correction. Integration of the corresponding protons from PEG (3.6 ppm) and methylene protons at (1.75 ppm) was used to quantify the number of ethylene units on the nanoparticles. Due to two different PEGs being used and no way of distinguishing the two by NMR, it was not possible to calculate the number of PEG chains on the nanoparticles.

### 2.3. Animal Studies

All animal experiments were performed using female athymic nude foxn1^nu^ mice. These studies were approved and performed according to the Norwegian Food Safety Authority (Permit number 13581) and conducted according to the regulations of the Federation of European Laboratory Animal Science Association (FELASA). The mice were kept under pathogen-free conditions, at constant temperature (21.5 ± 0.5 °C) and humidity (55 ± 5%), with 15 air changes/h and a 12 h light/dark cycle. They had access to food pellets and distilled water ad libitum; the water was supplemented with 17-β-estradiol at a 4 mg/L concentration [19]. Mice used for biodistribution were given a low fluorescent diet (2016S, Envigo, Madison, WI, USA) to avoid auto-fluorescence from food. All mice used (age 5–6 weeks and bodyweight of 18–20 g) were locally bred at the Department of Comparative Medicine, Oslo University Hospital, Norway. Anesthesia was obtained with 4% (*v*/*v*) Sevofluran (Baxter, Deerfield, IL, USA) along with 1 L/min oxygen and 3 L/min nitrous oxide in all the procedures where anesthesia was required.

The in-house established orthotopically growing basal-like xenograft MAS98.12 [19] was used as previously described [20]. The MAS98.12 tumors (pieces of 2–3 mm^3^) were implanted bilaterally into the mammary fat pad. The mice were inspected daily. When palpable, the tumors were measured by calipers to calculate the tumor volume according to the formula 0.5 × length × width^2^. When the tumors reached approximately 6 mm in diameter, the mice were randomly assigned to the different groups (the average volume of each group was 130–152 mm^3^).

The three different NR668-labeled PACA NPs were given as i.v. tail vein injections of 2.0 mg per animal to study biodistribution. For macrophage infiltration and MR spectroscopy studies, we injected 3.5 mg particles per animal of empty (not labeled with NR668) PBCA or PEBCA NPs, but due to toxicity issues, we injected only 0.5 mg POCA NPs per animal. The injection solutions were prepared directly before the administration by diluting the working solutions with 0.9% (*w*/*v*) NaCl. Mice were monitored daily for health status and were sacrificed by cervical dislocation at the end of the experiment or if the health condition deteriorated. The tumor volume was calculated at the end of the experiment.

### 2.4. In Vivo Imaging

PACA NPs, labeled with the fluorescent dye NR668, were used to study the biodistribution in MA98.12 bearing mice using an IVIS^®^ Spectrum in vivo imaging system (Perkin Elmer, Waltham, MA, USA). The best signal-to-noise ratio was obtained using the excitation/emission wavelength pair of 535/640 nm, and thus, this setting was used for the imaging of both whole body and organs. Whole-body images were obtained (not shown) 4, 24, and 48 h after injection (n = 2 for control and for each type of NPs at each time point); then, the animals were sacrificed by cervical dislocation, and organs were harvested and imaged ex vivo to measure fluorescence signaling. The relative signal intensity in the organs was calculated, using Living Image software (Perkin Elmer), as radiant efficiency (Emission light [photons/sec/cm^2^/str]/Excitation light [μW/cm^2^] × 10^9^) per pixel of the region of interest (drawn around the respective organs). The fluorescence intensity of the three PACA preparations was also measured using IVIS^®^ Spectrum with the same setting as described above.

### 2.5. Tumor Preparations Used for Immunohistochemistry and MR Spectroscopy

Tumors from MAS98.12 bearing mice were collected 24 and 96 h after injection of the empty PACA NPs. Tumors were split into two parts, one was immediately snap-frozen, and the other was preserved in 4% (*v*/*v*) formalin and used for immunohistochemistry. The frozen tumor tissues obtained following the injection of PEBCA or PBCA NPs were used for MR spectroscopy. The tumor samples, used for immunohistochemistry, were paraffin-embedded and sectioned into 3 µm consecutive slides.

### 2.6. Immunohistochemistry

The deparaffinization agent Neo-clear and mounting agent Neo-mount were obtained from VWR (Radnor, PA, USA). Heat-induced epitope retrieval was obtained by placing the deparaffinized slides in a water bath for 20 min at 100 °C with 10 mM sodium citrate buffer (pH 6.0). The slides were incubated with hydrogen peroxide solution provided with a HRP/DAB micro-polymer detection kit (ab236469, Abcam, Cambridge, CB, UK) for 10 min to block endogenous peroxidase activity. Then, sections were washed, and the blocking of non-specific binding was performed according to manufacturer’s protocol with protein block provided with ab236469 kit (Abcam).

Then, the sections were incubated for 60 min with the primary antibodies: anti-CD68 (1 mg/mL; ab125212, Abcam, Cambridge, CB, UK), anti-CD206 (0.1 µg/mL; ab64693, Abcam, Cambridge, CB, UK), and anti-iNOS (0.5 µg/mL; ab15323, Abcam, Cambridge, CB, UK) diluted in tris-buffered saline (TBS; 50 mM Tris-Cl, 150 mM NaCl, pH 7.6) solution with 1% bovine serum albumin (BSA). Negative control sections were stained without using primary antibodies. CD68 is a normally used marker for the whole macrophage population [21,22], whereas iNOS (inducible nitric oxide synthase) is a common marker for M1 macrophages (anti-tumorigenic and pro-inflammatory macrophages) [23], and CD206 is a marker for M2 macrophages (pro-tumorigenic and anti-inflammatory macrophages) [23]. Washing, between the steps, was performed using TBS, and the primary antibodies were detected using the HRP/DAB micro-polymer detection kit as described by the manufacturer (ab236469, Abcam). The slides were incubated with the chromogen solutions provided with the ab236469 kit (Abcam) and counterstained using hematoxylin and 37 mM ammonium hydroxide containing solution (Sigma-Aldrich, St. Louis, MO, USA).

The stained tissue sections were scanned (NanoZoomer HT, Hamamatsu Photonics, Shizuoka, Japan), using a 40× objective. The relative amount of staining of CD68, iNOS, and CD206 was automatically scored, using the ImmunoPath software (Room4 Ltd., Crowborough, ES, UK) [24]. In order to obtain a correct quantification, the tumor areas were annotated manually, excluding necrotic areas and blood vessels. Then, the annotated areas were broken down to smaller tiles per image of interest for effective processing and were analyzed further by computerized image analysis. The analysis protocol was developed using different tumor images, which represented the staining variation for each antibody. The optimal color ranges for the positive pixels were specified based on hue, saturation, and value thresholds as described [25]. ImmunoPath reported the number of positive pixels, and the size of the tumor area to calculate positive pixels/whole image area. Four tumors from each treatment were extracted after 96 h for immunohistochemistry analyses. We decided to remove one data point for iNOS analyses following injection of PEBCA NPs. This data point was more than 80 times (81–98) lower than the three other data points for this analysis. We decided to keep the data for this tumor for CD206 and CD68, as all data points for these analyses were very similar.

### 2.7. MR Spectroscopy

Tumor tissue was cut to fit into a 30-μL disposable insert containing a lock reference (25 mM formate in D_2_O), locked tightly in a 4 mm zirconium rotor. The HR MAS MR spectra were recorded using a Bruker Advance DRX600 (14.1 T) spectrometer (Bruker Biospin GmbH, Rheinstetten, Germany) containing a ^1^H/^13^C MAS probe. Samples were spun at 5 kHz at a magic angle, and the temperature was kept at 4 °C throughout the experiment. NMR spectra were acquired using a one-dimensional ^1^H NOESY pulse sequence with water presaturation (Bruker; noesygppr1d) as described [26]. A total of 256 scans were recorded per sample. After the automatic phase and baseline correction, spectra were imported into Matlab (The Mathworks, Inc., Narick, MA, USA) for multivariate analysis in PLS Toolbox (Eigenvector Research Inc., Manson, WA, USA). Principal component analysis (PCA) was performed after peak alignment and normalization of samples to the total area under the curve.

### 2.8. Statistical Analyses

For quantification of the fluorescence signal in the biodistribution studies, the mean of the two data points was calculated. For quantification of positive pixels in the immunohistochemistry studies, multiple *t*-tests, corrected for multiple comparisons using the Holm–Sidak method was performed. The statistical analyses were done using GraphPad Prism (version 8.0.1 for Windows, GraphPad Software, San Diego, CA, USA).

## 3. Results and Discussion

### 3.1. Characterization of PACA Particles

All nanoparticles used in this study were similar with regard to size, polydispersity index (PDI), and zeta potential, as listed in Table 1. NMR showed that the number of ethylene units per nm^2^ on the particles was similar for fluorescently labeled PBCA and POCA, whereas PEBCA particles had approximately 40% more ethylene units. The PEBCA NP was also the one with the least protein adsorption as determined by size increase when mixed with human plasma.

### 3.2. Biodistribution of NR668 Loaded PBCA, PEBCA and POCA NPs in Mice

**Figure 1:**
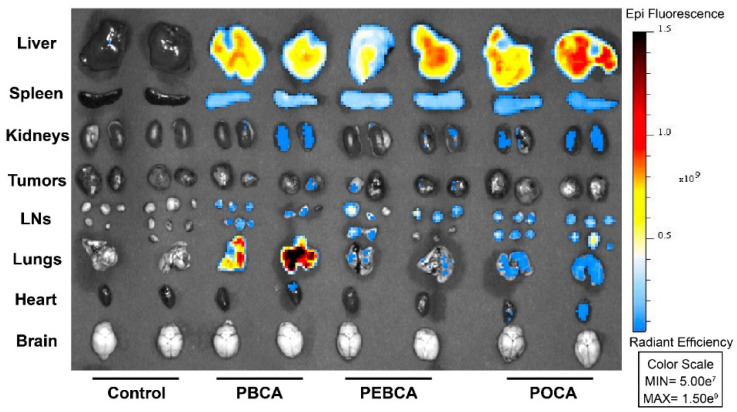
Biodistribution of PACA NPs containing the fluorescent dye NR668 in mice bearing MAS98.12 tumors. Organs were removed 24 h after i.v. injection. In vivo imaging system (IVIS) was used to image liver, spleen, kidneys, tumors, lymph nodes (LNs), lungs, heart, and brain. The color scale to the right indicates radiant efficiency. Control animals did not receive any treatment.

**Figure 2:**
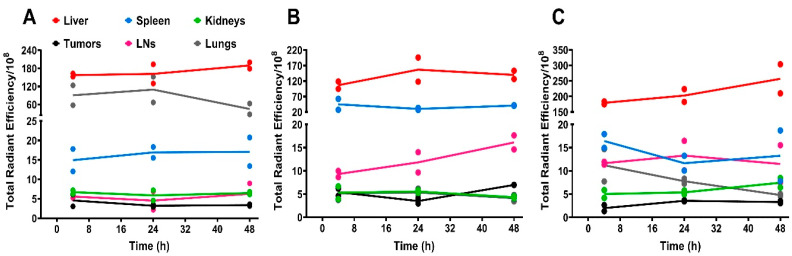
Quantification of fluorescence intensity as relative radiant efficiency/10^8^ pixel data of organs in MAS98.12 tumor bearing mice collected at different time points after the injection of NR-668-loaded PACAs. Quantification of fluorescence intensity based on treatment with PBCA (**A**), PEBCA (**B**), and POCA (**C**). Data plotted for liver (red), spleen (blue), kidneys (green), tumors (black), lymph nodes (pink), and lungs (gray). Since the fluorescence intensity was somewhat different for the three preparations, this is corrected for by multiplying the PBCA values with 0.91 and the POCA values with 0.79. PBCA: poly(butyl cyanoacrylate), PEBCA: poly(2-ethyl-butyl cyanoacrylate), and POCA: poly(octyl cyanoacrylate). Lines connect the mean of the two individual data points. LNs: lymph nodes.

**Figure 3:**
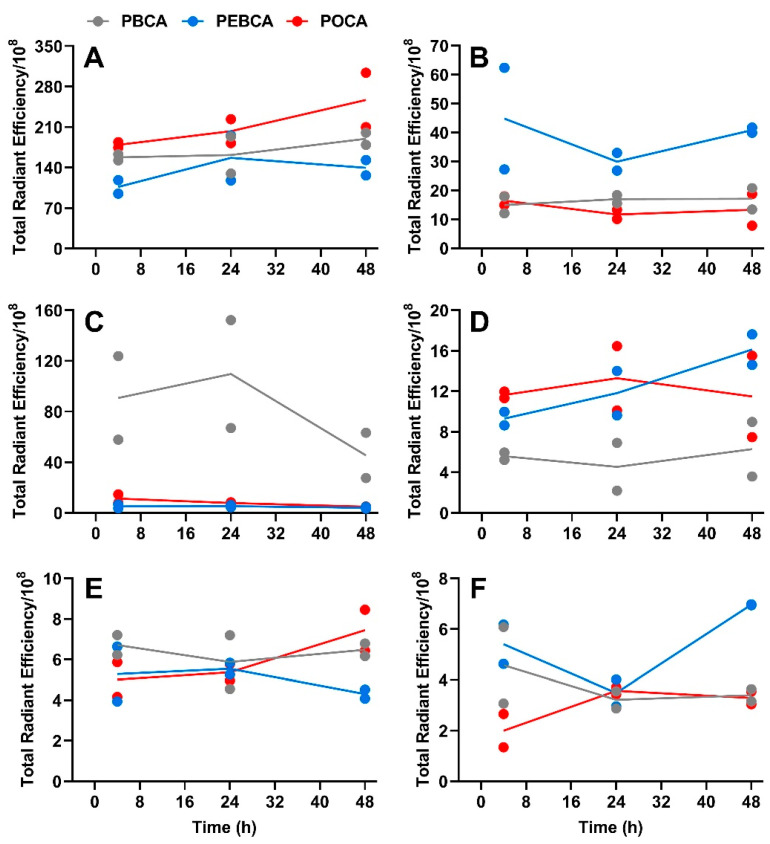
Quantification of fluorescence intensity as relative radiant efficiency/10^8^ pixel data of organs in MAS98.12 tumor-bearing mice collected at different time points after the injection of NR668-loaded PACAs. The fluorescence intensity data for the three PACAs are compared for the various tissues, i.e., in liver (**A**), spleen (**B**), lungs (**C**), lymph nodes (**D**), kidneys (**E**), and tumors (**F**); PBCA (gray), PEBCA (blue), and POCA (red). Since the fluorescence intensity was somewhat different for the three preparations, this is corrected for by multiplying the PBCA values with 0.91 and the POCA values with 0.79. Lines connect the mean of the two individual data points.

The biodistribution of the three NR668-labeled PACA NPs was studied in MAS98.12 bearing mice by fluorescence imaging up to 48 h after i.v. injection of 2 mg NPs. The IVIS^®^ Spectrum scanner was used to image mice at 4, 24, and 48 h (not shown); then, the mice were sacrificed such that organs could be harvested and visualized ex vivo. Images of organs/tissues (liver, spleen, kidneys, tumors, lymph nodes, lungs, heart, and brain) harvested 24 h after injections are shown in Figure 1. The mean radiant efficiency relative to the pixel size of the region of interest per organ was quantified and is plotted in Figure 2A–C and Figure 3A–F (data for brain and heart were close to the detection limit and are thus not included). The data shown in Figure 2 and Figure 3 are corrected for the differences measured in the fluorescence intensity of the three PACA preparations. The data used in Figure 2 and Figure 3 are from the same experiments. The two figures are presented separately to demonstrate the quantification of biodistribution for each of the NPs (Figure 2) and for each organ (Figure 3). As reported earlier [15], injection of the free NR668 dye did not give any detectable fluorescence with the wavelengths used. Together with our earlier report showing that NR668 did not leak from NPs with a similar composition [27], these data indicate that the detected signals are from PACA-bound NR668 and not from free/released NR668.

The curves shown in Figure 3 reveal a high uptake of all the three PACAs in liver (Figure 3A) and spleen (Figure 3B). This is in line with our earlier studies with PEBCA NPs [15,28], and similar to what is reported for many other NPs [29,30,31]. All the PACA NPs were readily detectable in all organs/tissues, including lungs (Figure 3C), lymph nodes (Figure 3D), kidneys (Figure 3E), and tumors (Figure 3F). These data also demonstrate a very different biodistribution of the three PACAs, despite that they are made by the polymerization of monomers with very similar chemical structures and that they are similar in size and charge (Table 1). Surprisingly, the PBCA NPs with a hydrodynamic diameter of 167 nm showed a very high recovery in lungs, which was much higher than obtained with the other two PACA NPs. We earlier reported that the PBCA NPs also deviated from PEBCA and POCA NPs by giving the clustering of lysosomes in cells [11], but so far, we do not know why the PBCA NPs distribution differed from the other PACA NPs. As shown earlier [15], the PEBCA NPs showed an accumulation in lymph nodes (Figure 3D). This suggests a clinically relevant potential for the treatment of tumors disseminated to lymph nodes [32,33]. Lymph nodes are not only the first site of breast cancer metastasis but also a well-recognized distant metastatic site, particularly in the triple negative basal-like breast cancer [34]. It should also be noted that the PEBCA NPs demonstrated the highest recovery of the NPs in the primary tumor site (the MAS98.12 tumors) at 48 h after injection (Figure 3F). It is not possible to quantify the percent of the injected dose of NPs accumulating in tumors when using the IVIS^®^ fluorescent imaging. The fluorescence signal observed in the tumors is low and thus in agreement with the data in the review by Wilhelm et al. [35], who reported a mean tumor accumulation of 0.7% of the injected dose of various NPs from 200 studies using different tumor models.

Discussions about how the pharmacokinetics and biodistribution depend on the size and charge of NPs have been ongoing for many years. These characteristics are often discussed in light of the density, chain lengths, and other properties of attached PEG-containing molecules, with a particular focus on how the PEG chains affect the binding of proteins (i.e., the protein corona) to the NPs (see e.g., [29,30,31,36,37,38,39,40,41,42,43,44]). The protein corona described in these studies often consists of proteins involved in complement activation, blood coagulation, macrophage uptake, and lipid metabolism. There are many speculations about how these proteins can contribute to differences in pharmacokinetics, biodistribution, cellular uptake, and biological effects of NPs. Notably, it has been reported that there are differences between the protein corona obtained in vitro (mice plasma) and in vivo in mice [45]. Thus, more studies are needed to investigate whether in vitro protein binding data of NPs can explain their in vivo behavior. Uptake of NPs by the residential liver and spleen macrophages, i.e., uptake by the mononuclear phagocytic system (MPS) earlier often called the reticuloendothelial system (RES), is frequently reported to account for the removal from circulation of most NPs in vivo [29,30,31,43,44]. This includes the quantitative biodistribution study in humans of radioactively labeled liposomes similar to Doxil^®^/Caelyx^®^ [46].

It is possible that different proteins associated with the three PACA NPs studied here may contribute to the observed differences in biodistribution. However, as discussed above, we are far from understanding how the protein coronas may contribute to such large variations. We find it especially surprising to see such large differences in biodistribution with our three PACA NPs, with a similar size distribution, charge, and number of ethylene units on the surface (Table 1).

### 3.3. Infiltration of Macrophages into the MAS.98.12 Tumors

**Figure 4:**
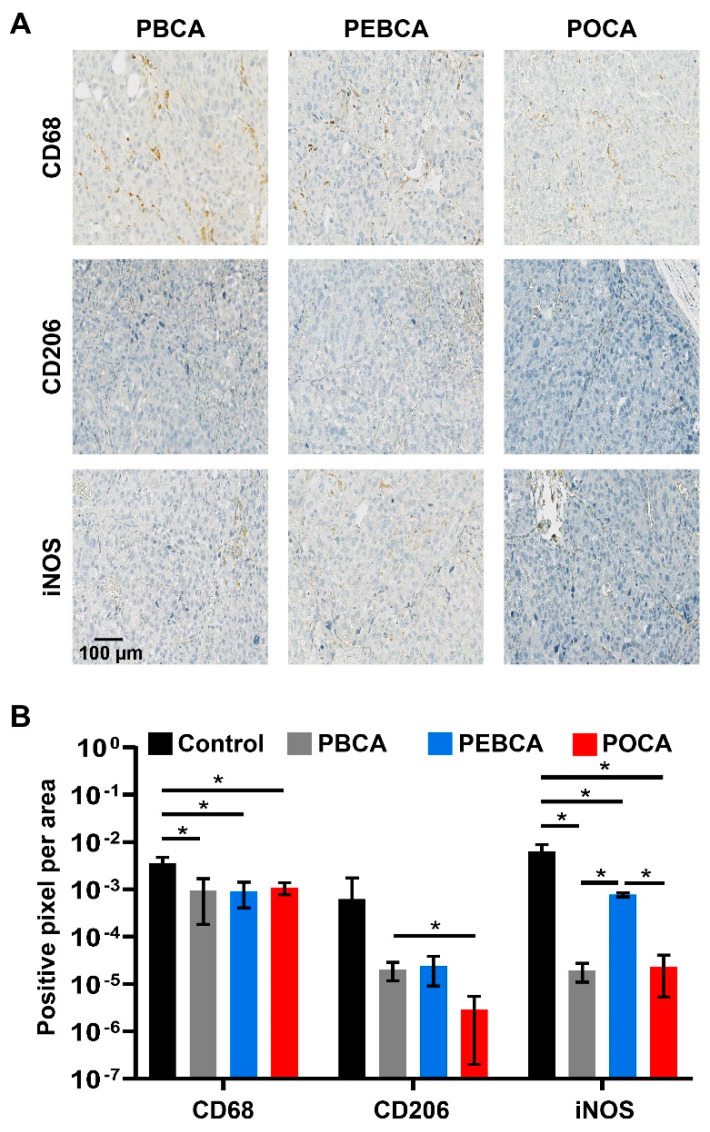
Macrophage infiltration into MAS98.12 tumors in mice measured 96 h after i.v. injection of PACA NPs. Representative images of PACA NPs treated tumors with different macrophage markers are presented (**A**). The total population of infiltrated macrophages was estimated using an antibody against CD68. The population of pro-tumorigenic (M2, anti-inflammatory) was estimated using an antibody against CD206, and the population of anti-tumorigenic (M1, pro-inflammatory) macrophages was estimated using an antibody against iNOS. Quantification of infiltrated macrophages after treatment with PBCA, PEBCA, or POCA, or without treatment (control) is plotted (**B**). Data are presented as mean ± SD (n = 4). Asterisks indicate statistical significance in the level of phenotype-specific macrophages between untreated tumors or tumors treated with the different NPs using multiple *t*-tests (Holm–Sidak method), where *p* < 0.05 is marked with *. PBCA: poly(butyl cyanoacrylate), PEBCA: poly(2-ethyl-butyl cyanoacrylate), POCA: poly(octyl cyanoacrylate).

Macrophages are the most abundant immune cells in tumors [47]. The tumor-associated macrophages (TAMs) were earlier believed to exert anti-tumor activities. However, later experimental data and clinical experience demonstrate that TAMs may influence anticancer drug responses and also promote tumor progression [48,49,50]. The M2-type macrophages are known as alternatively activated macrophages and referred to as pro-tumorigenic or anti-inflammatory, whereas the classically activated pro-inflammatory macrophages (M1) have anti-tumorigenic properties [51,52]. Dynamic changes in the macrophage population define the different subtypes [48], and a set of markers is usually recommended for a comprehensive characterization of the macrophage population [23,53]. In the present study, we have used the well-accepted CD68 as a marker for the total macrophage population, nitric oxide synthase (iNOS) for detection of the M1 phenotype, and mannose receptor (CD206) for detection of the M2-phenotype (Figure 4A) [23,53].

The infiltration of total macrophages and the M1 and M2 subpopulations into MAS98.12 tumors were assessed using automatic quantification of digitized slides obtained 96 h after i.v. injections of PBCA, PEBCA, and POCA NPs. The data in Figure 4B show a similar infiltration of total macrophages (CD68 positives) in all the treatments, whereas significantly more iNOS stained macrophages (M1, anti-tumorigenic, pro-inflammatory) were present in tumors treated with PEBCA NPs than with PBCA or POCA NPs. Thus, also, these data indicate that it may be beneficial to use PEBCA NPs compared to PBCA or POCA NPs for the delivery of drugs to tumors. Staining with the CD206 antibody showed less M2 macrophages (anti-inflammatory, pro-tumorigenic) present in the tumors following injection of POCA NPs than the two other NPs. However, the higher toxicity observed for the POCA NPs in mice makes these NPs less promising for in vivo use, despite that they result in less tumor infiltration of pro-tumorigenic macrophages. It should be noted that increasing the ratio of M1/M2 macrophages as observed for the PEBCA NPs in the present study has been reported to have promising therapeutic effects in other cancer models in mice [54]. The increased ratio of M1/M2 macrophages in the tumors after the injection of PEBCA NPs is in agreement with our previous observation [15], where we compared macrophage infiltration following the injection of CBZ-loaded PEBCA NPs, empty PEBCA NPs, and free CBZ.

The M2 macrophages have been reported to demonstrate a strong endocytic uptake by macropinocytosis; in contrast, such an uptake was almost absent in the M1 macrophages [55]. We speculate that the high endocytic uptake by the M2 macrophages combined with the higher cytotoxicity of POCA than PEBCA and PBCA NPs [11] explains why fewer M2 macrophages are observed in the MAS98.12 tumors following injection of POCA NPs. Interestingly, pro-tumorigenic M2 macrophages have been reported to use endocytosis to degrade collagen and promote tumor growth in solid tumors [54]. It is still unknown why the three PACA NPs differ in their effects on the ratio of infiltrated M1/M2 in the MAS98.12 tumors, although we speculate that differences in the protein corona may be of importance.

### 3.4. HR MAS MR Spectroscopy of Tumors

**Figure 5:**
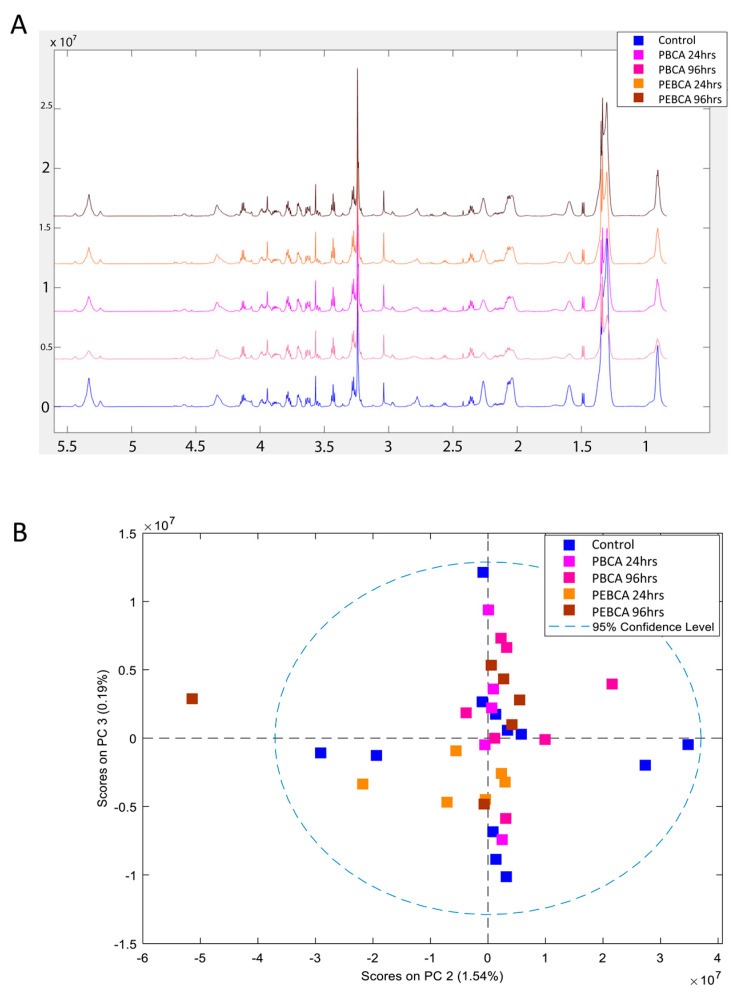
High-resolution magic angle spinning magnetic resonance (HR MAS MR) spectroscopy of tumor tissue. The metabolic fingerprints of tumor tissue from untreated controls and tumor tissue exposed to PEBCA and PBCA were highly similar (**A**). The PCA analysis did not indicate any significant differences between the treatment groups (**B**). PBCA: poly(butyl cyanoacrylate), PEBCA: poly(2-ethyl-butyl cyanoacrylate).

The metabolic profiles obtained using HR MAS MR spectroscopy to analyze tumor slices following injection of PBCA and PEBCA NPs looked very similar (Figure 5A). PCA analysis did not reveal any differences in these spectra (Figure 5B).

We have earlier used HR MAS MR spectroscopy to demonstrate the distinct metabolic fingerprint of the MAS98.12 xenograft model and metabolic responses to drug therapy [56,57]. However, the present data show that the difference in macrophage subpopulations or other changes due to the presence of the PBCA or PEBCA NPs was insufficient to perturb the overall metabolic characteristics of the tumors.

## 4. Conclusions

In summary, biodistribution studies demonstrated a promising tumor tissue distribution, in addition to an enhanced accumulation in lymph nodes, of the PEBCA NPs compared to the two other PACA NPs. Injection of the PEBCA NPs led to an increased ratio of M1/M2 macrophages in the MAS98.12 tumors, similarly to what has been reported to give promising therapeutic effects in other cancer models in mice. This increased ratio of M1/M2 is exciting and should be further explored as it might suggest that the PEBCA NPs may modulate the macrophage composition and contribute to enhance the therapeutic effect of the encapsulated drugs.

## Figures and Tables

**Table 1:** Description of size, polydispersity index (PDI), zeta potential, amount of polyethylene glycol (PEG), and NR668 fluorescence of the PACA NPs batches used in this study. PBCA: poly(butyl cyanoacrylate), PEBCA: poly(2-ethyl-butyl cyanoacrylate), POCA: poly(octyl cyanoacrylate).

Nanoparticle Preparations	DLS: Size z-avg (nm)	DLS: Size n-Mean (nm)	DLS: PDI	Zeta-Potential (mV)	NTA ^a^: Size n-Mean (nm)	NTA ^a^: Size n-Mean in Plasma (nm)	NR668 Content ^b^	Ethylene Units/nm^2^	Structure of Monomers (BCA, EBCA, OCA)
PBCA	187	142	0.17	−2.5	-	-	-	-	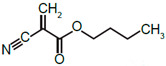
PBCA-NR668	149	139	0.12	−2.6	140	176	0.91	12.7	
PEBCA	155	123	0.08	−2.7	-	-	-	-	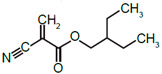
PEBCA-NR668	193	150	0.13	−2.2	183	207	1.0	16.8	
POCA	184	134	0.19	−2.1	-	-	-	-	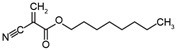
POCA-NR668	175	159	0.10	−2.9	169	197	0.79	11.4	

**^a^** Measured in fluorescence mode. **^b^** Content of NR668 (fluorescence) relative to that measured with the PEBCA NPs. PBCA: poly(ethyl cyanoacrylate), PEBCA: poly(2-ethyl-butyl cyanoacrylate), POCA: poly(octyl cyanoacrylate).

## Data Availability

The data presented in this study are available within this article. Further inquiries may be directed to the authors.

## Abbreviations

AUC: area under the curve; EPR: enhanced permeability and retention; i.v.: intravenous; LNs: lymph nodes; PACA: poly(alkyl cyanoacrylate); PBCA: poly(n-butyl cyanoacrylate); PDI: polydispersity index; PDX: patient-derived xenograft; PEBCA: poly(2-ethyl-butyl cyanoacrylate); PEG: polyethylene glycol; POCA: poly(octyl cyanoacrylate); NPs: nanoparticles; TAMs: tumor-associated macrophages.
